# Predictive factors for a long-term response duration in non-squamous cell lung cancer patients treated with pemetrexed

**DOI:** 10.1186/s12885-016-2457-0

**Published:** 2016-07-07

**Authors:** Sojung Park, Hyun Jung Kim, Chang–Min Choi, Dae Ho Lee, Sang–We Kim, Jung–Shin Lee, Woo Sung Kim, Se Hoon Choi, Jin Kyung Rho, Jae Cheol Lee

**Affiliations:** Department of Pulmonary and Critical Care Medicine, University of Ulsan, College of Medicine, Asan Medical Center, 388-1 Pungnap–Dong, Songpa-gu, Seoul, 05505 South Korea; Department of Internal Medicine, Kyungpook National University Hospital, Kyungpook National University School of Medicine, 130 Dongdeok-ro, Jung-gu, Daegu, 41944 South Korea; Department of Oncology, University of Ulsan, College of Medicine, Asan Medical Center, 388-1 Pungnap–Dong, Songpa-gu, Seoul, 05505 South Korea; Department of Thoracic and Cardiovascular Surgery, University of Ulsan, College of Medicine, Asan Medical Center, 388-1 Pungnap–Dong, Songpa-gu, Seoul, 05505 South Korea; Asan Institute for Life Sciences, Asan Medical Center, 388-1 Pungnap–Dong, Songpa-gu, Seoul, 05505 South Korea

**Keywords:** Pemetrexed, Non-small-cell lung cancer, Prognosis, Progression-free survival, EGFR, Anaplastic lymphoma kinase

## Abstract

**Background:**

Pemetrexed is widely used for the treatment of advanced non-squamous non-small-cell lung cancer (NSCLC). However, factors that can predict the benefits of pemetrexed therapy have not yet been defined.

**Methods:**

We compared the clinical and molecule pathological characteristics of good and poor responders among a cohort of 1,848 non-squamous NSCLC patients who had received at least two cycles of pemetrexed therapy between November 2006 and February 2015. Among these cases, 92 good responders who were the top 5 % in terms of progression-free survival (PFS) and 222 poor responders who had progressive disease after only 2 cycles of therapy were selected for the analysis.

**Results:**

The median PFS of the good responders was 29.9 months (range; 20.9–90.0) and the median number of cycle was 37 (range; 18–129). Although 53.5 % of patients showed stable disease (SD), this response was sustained (median PFS in SD, 29.6 months). A never-smoking status was related to better survival outcome, whereas *EGFR* mutation, two or more metastatic sites, and intra-abdominal metastasis were each associated with a poor PFS. ALK translocation showed a tendency for a positive impact on response to pemetrexed, whereas metastatic lesion to liver, adrenal gland or bone showed a tendency for a negative impact despite not reaching our threshold for statistical significance.

**Conclusions:**

Predictive factors, such as smoking status, the status of genetic alteration and tumor burden, should be considered when administering pemetrexed therapy for non-squamous NSCLC.

## Background

Lung cancer is the leading cause of death worldwide. In Korea, 40.6 % of lung cancer patients have a metastatic lesion at the time of diagnosis [1]. Despite progress in the understanding of cancer biology and development of new therapeutic agents, the 5-year total survival rate for lung cancer remains 19.7 % overall and drops to 4.8 % in patients with a metastatic lesion at the time of diagnosis [1]. Therefore, optimal treatments to improve the outcomes of patients with advanced lung cancer are still needed.

Pemetrexed is a multi-targeting antifolate that can inhibit thymidylate synthase (TS) and other folate-dependent enzymes that are involved in purine and pyrimidine synthesis. Pemetrexed has been widely used to treat patients with non-squamous non-small-cell lung cancer (NSCLC) because two separate phase III trials of this drug have reported prolonged survival in patients with non-squamous cell carcinoma compared with those with squamous cell carcinoma [2, 3]. Other than histologic diagnoses, some factors related to the efficacy of pemetrexed have been suggested. For example, sex was found to have a prognostic impact on survival in a phase III trial [4]. Additionally, never-smoker, anaplastic lymphoma kinase (*ALK*) gene rearrangement, low tumor TS RNA level, thyroid transcription factor-1 (TTF-1) expression and low serum leptin level were associated with a good response to pemetrexed in previous studies [4–10]. However, because most earlier studies did not primarily focus on the pemetrexed response, we believe that more investigations aimed at identifying factors that correlate with an improved outcome after pemetrexed therapy are needed.

## Methods

### Study population

Since pemetrexed was approved for the treatment for non-squamous NSCLC in Korea at November 2006, we identified non-squamous NSCLC patients who received a pemetrexed-containing regimen between November 2006 and February 2015 at Asan medical center, Seoul, South Korea. Patients were arranged in order of the duration of pemetrexed therapy. Among these cases, patients who had been given pemetrexed once were excluded, because they were more likely to be lost to follow-up for several reasons other than experiencing rapid progression of the disease. Most of these patients were transferred to hospice facility or rejected to receive further chemotherapy after initiation of pemetrexed therapy. Since we intended to evaluate the efficacy of pemetrexed in the current study, we selected patients who received two or more cycles of pemetrexed and underwent follow-up imaging study. Additionally, there was a strong suspicion that some of those individuals had been given pemetrexed despite showing a poor performance status, due to the high tolerability and low toxicity of this drug; however, those patients eventually should have been discontinued treatment because of their performance status [11]. We also excluded patients for whom treatment was discontinued for reasons other than disease progression, such as a poor performance status, infection, transfer to another hospital, and dropout. Finally, of the remaining patients, good responders (top 5 percent) and poor responders (bottom 5 percent) were planned to be selected for comparison in our present analyses. The study design was approved by the Institutional Review Board of Asan Medical Center, which waived the requirement for informed consent due to the retrospective nature of the analysis.

### Baseline and treatment assessments

We retrospectively reviewed clinicopathological data and follow-up information contained in the archived medical records in April 2015. The date of data cutoff was February 28, 2015. Tumor histology was classified by pathologists using the standard World Health Organization criteria. We have examined the expression of TTF-1 using immunohistochemistry (1:200 dilution; Novocastra Lab., Newcastle, UK). *ALK* status was determined by the Vysis *ALK* Break Apart FISH probe kit (Abbott Molecular, Inc., Abbott Park, IL, USA). We analyzed epidermal growth factor receptor (*EGFR*) mutations within exons 18 to 21 and Kirsten rat sarcoma viral oncogene homolog (*KRAS*) mutations by a direct DNA sequencing method using an automatic ABI PRISM 3100 Genetic Analyzer (Applied Biosystems, Foster City, CA, USA). Patients were administered pemetrexed alone or in combination with platinum or non-platinum agents at a dose of 500 mg/m^2^ every 3 weeks. Maintenance pemetrexed therapy after 4 cycles of pemetrexed-platinum was considered as combination therapy. To evaluate tumor responses, chest radiography was performed during every cycle, and chest computed tomography was performed every 2 to 3 cycles. If a patient was suspected to have a new extrapulmonary lesion, we immediately performed additional procedures, such as abdominal computed tomography, magnetic resonance imaging, bone scintigraphy, and positron emission tomography. Progression-free survival (PFS) was defined as the time from pemetrexed commencement to either documented disease progression or death from any cause. For patients without evidence of disease progression at the date of data cutoff, patients in the good responder group were censored at the time of data cutoff. Disease status was assessed based on the Response Evaluation Criteria in Solid Tumors version 1.1 [12].

### Statistical analysis

Categorical variables were analyzed using either Pearson’s chi-square test or Fisher’s exact test and continuous variables were analyzed using either Student *t*-test or Mann–Whitney test. Variables selected by univariate analysis (*p* <0.1) were evaluated in a multivariate analysis using the Cox proportional hazard model. All tests for significance were two-sided, and all variable with a *p*-value <0.05 were considered to be significant. All statistical analyses were performed with SPSS software version 21.0 (SPSS Inc., Chicago, IL, USA).

## Results

### Patient characteristics

**Fig. 1:**
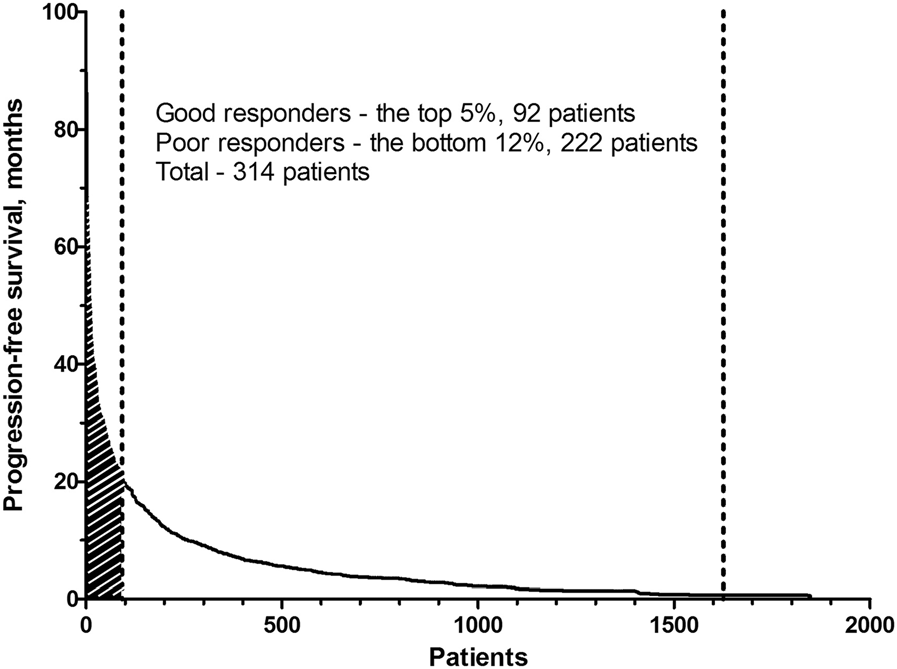
Distribution of non-small-cell lung cancer patients. Distribution of non-small-cell lung cancer patients arranged in order of the duration of pemetrexed therapy. The dotted lines indicate the cutoff for the top 5 % and bottom 12 % of patients

From November 2006 to February 2015, a total of 2,310 non-squamous NSCLC patients received pemetrexed-containing chemotherapy. Among these patients, 462 patients met the exclusion criteria. Of the remaining 1,848 patients, the bottom 14 % (252 patients) discontinued treatment after receiving two cycles of pemetrexed-containing treatment because of disease progression in 222 cases, transfer to another hospital in 16 cases, a poor performance status in 7 cases, and pneumonia in another 7 cases. As pemetrexed was given in same cycle to those bottom 12 % of overall patients, a final cohort included 314 patients; the top 5 % (92 patients) and the bottom 12 % (222 patients) (Fig. 1). We termed these patient groups as good responders and poor responders, respectively.

The baseline demographic and clinical characteristics of the patients are presented in Table 1. The mean age of the 314 patients was 59.1 ± 10.1 years old. Female (*P* = 0.009) and never smokers (*P* = 0.015) were significantly predominant in the good responder group compared with the poor responder group. Among the 314 patients, 263 (83.8 %) received pemetrexed alone, 34 (10.8 %) received pemetrexed in combination with a platinum agent, and 17 (5.4 %) received pemetrexed in combination with a nonplatinum agent. The number and types of regimens used prior to pemetrexed were not significantly different between groups.

**Table 1:** Baseline patient characteristics

	Total (*n* = 314)	Good responders (*n* = 92)	Poor responders (*n* = 222)	*P-*value
Age, years, mean ± SD	59.1 ± 10.1	59.2 ± 10.1	59.1 ± 10.1	0.953
Sex, Female	145 (46.2)	53 (57.6)	92 (41.4)	0.009
History of smoking (*n* = 303)	303	86	217	0.015
Current smoker	68 (22.4)	12 (14.0)	56 (25.8)	
Ex-smoker	76 (25.1)	18 (20.9)	58 (26.7)	
Never smoker	159 (52.5)	56 (65.1)	103 (47.5)	
Stage				0.456
≤IIIB	19 (6.1)	7 (7.6)	12 (5.4)	
IV	295 (93.9)	85 (92.4)	210 (94.6)	
Stage M1a	93 (29.6)	44 (47.8)	49 (22.1)	<0.001
Stage M1b	202 (64.3)	41 (44.6)	161 (72.5)	<0.001
Type of chemotherapy				0.002
Pemetrexed monotherapy	263 (83.8)	68 (73.9)	195 (87.8)	
Pemetrexed combination	51 (16.2)	24 (26.1)	27 (12.2)	0.552
Platinum	34 (10.8)	15 (16.3)	19 (8.6)	
Non-platinum	17 (5.4)	9 (9.8)	8 (3.6)	
Previous chemotherapy				0.068
0	44 (14.0)	18 (19.6)	26 (11.7)	
≥1	270 (86.0)	74 (80.4)	196 (88.3)	
Previous regimen (*n* = 270)^a^	270	74	196	
Response to Gemcitabine^b^	88/175 (50.3)	32/49 (65.3)	56/126 (44.4)	0.013
Response to EGFR-TKI	79/128 (61.7)	16/34 (47.1)	63/94 (67.0)	0.040
Response to Paclitaxel	36/56 (64.3)	5/14 (35.7)	31/42 (73.8)	0.010
Response to Docetaxel	7/14 (50.0)	4/6 (66.7)	3/8 (37.5)	0.592
Response to Miscellaneous	7/15 (46.7)	3/4 (75.0)	4/11 (36.4)	0.282

Data are shown as n (%) per each group, unless otherwise noted

^a^Total number exceeds 270 as some patients had received two or more regimen prior to pemetrexed

^b^Response was defined as achievement of partial response or stable disease for 3 months or more. Others were defined as non-response

*SD* standard deviation, *EGFR-TKI* epidermal growth factor receptor-tyrosine kinase inhibitor

### Efficacy

All patients in the poor responder group experienced disease progression after receiving two cycles of pemetrexed-containing therapy, and their median PFS was 1.4 months (range, 0.7–2.8). By contrast, the median PFS of the good responder group was 29.9 months (range, 20.9–90.0) and the median cycle number was 37 (range, 18–129). Additionally, 46.7 % of the good responders experienced a partial response and 53.3 % of them exhibited a stable disease.

### Histology and gene alterations

The histological and genetic disease characteristics are presented in Table 2. Most of the patients (285/314, 90.8 %) had adenocarcinoma and 24 patients (7.6 %) had NSCLC-not otherwise specified. Among the 57 cases in which the histological subtypes were identified, mucinous adenocarcinoma (22/57, 38.6 %) was the most frequent type, followed by acinar (13/57, 22.8 %) and papillary (13/57, 22.8 %) types. Some characteristics of good responders, such as the level of differentiation, TTF-1 expression and gene alterations, were significantly different from those of poor responders. A total of 46.1 % of poor responders had *EGFR* mutation, while 16.7 % of good responder had *EGFR* mutation (*P* <0.001). In contrast to *EGFR* mutation, *ALK* translocation was more frequently seen in the good responder group (22.2 % versus 4.2 %, *P* <0.001).

**Table 2:** Histological and genetic characteristics of the patients treated with a pemetrexed-containing therapy

	Total (*n* = 314)	Good responders (*n* = 92)	Poor responders (*n* = 222)	*P-*value
Histology				0.810
Adenocarcinoma	285 (90.8)	83 (90.2)	202 (91.0)	
Large cell carcinoma	5 (1.6)	2 (2.2)	3 (1.4)	
NSCLC-NOS	24 (7.6)	7 (7.6)	17 (7.7)	
Subtype (*n* = 57)*	57	24	33	0.368
Mucinous	22 (38.6)	10 (41.7)	12 (36.4)	
Acinar	13 (22.8)	7 (29.2)	6 (18.2)	
Papillary	13 (22.8)	4 (16.7)	9 (27.3)	
Signet-ring	5 (8.8)	3 (12.5)	2 (6.1)	
Solid	4 (7.0)	0	4 (12.1)	
Differentiation (*n* = 220)	220	63	157	0.001
Well-differentiated	18 (8.2)	12 (19.0)	6 (3.8)	
Moderately-differentiated	115 (52.3)	29 (46.0)	86 (54.8)	
Poorly-differentiated	87 (39.5)	22 (34.9)	65 (41.4)	
TTF-1 expression (*n* = 151)	125 (82.8)	35 (94.6)	90 (78.9)	0.028
Gene mutation (*n* = 239)	239	72	167	
* EGFR* mutation	89 (37.2)	12 (16.7)	77 (46.1)	<0.001
* ALK* translocation	23 (9.6)	16 (22.2)	7 (4.2)	<0.001
* KRAS* mutation	5 (2.1)	0	5 (3.0)	0.326
Wild-type for *EGFR/ALK/KRAS*	122 (51.0)	44 (61.1)	78 (46.7)	0.041

Data are shown as n (%) per each group, unless otherwise noted

*The subtype was not subclassified according to the International Association for the Study of Lung Cancer/American Thoracic Society/European Respiratory Society Classification of Lung adenocarcinoma revised at 2011, for study subjects were recruited from 2006

*NSCLC-NOS* Non-small-cell lung cancer-not otherwise specified; *TTF-1* thyroid transcription factor-1; *EGFR* epidermal growth factor receptor; *ALK* anaplastic lymphoma kinase; *KRAS* Kirsten rat sarcoma viral oncogene homolog

### Tumor burden and site of metastasis

The median number of metastatic sites was 1 (range, 0–5) in good responders and 2 (range, 0–7) in poor responders. Consequentially, patients with two or more metastatic sites were significantly more likely to be included in the poor responder group (*P* <0.001). The contralateral lung was the most common metastatic site, followed by the pleura, bone, brain, adrenal gland and liver. The poor responders had significantly more distant metastases other than contralateral lung or pleura, compared with the good responders (72.5 % versus 44.6 %, *P* <0.001). A total of 21.7 % of patients had undergone previous surgical therapy, including 40.2 % of the good responders and 14.0 % of the poor responders (*P* <0.001) (Table 3).

**Table 3:** Number and site of metastases in the patients treated with a pemetrexed-containing therapy

	Total (*n* = 314)	Good responder (*n* = 92)	Poor responder (*n* = 222)	*P-*value
Number of metastatic sites ≥2	201 (64.0)	44 (47.8)	157 (70.7)	<0.001
Site of metastases				
Lung	152 (48.4)	48 (52.2)	104 (46.8)	0.390
Pleura	144 (45.9)	39 (42.4)	105 (47.3)	0.427
Bone	125 (39.8)	29 (31.5)	96 (43.2)	0.053
Brain	77 (24.5)	18 (19.6)	59 (26.6)	0.189
Adrenal gland	45 (14.3)	3 (3.3)	42 (18.9)	<0.001
Liver	43 (13.7)	5 (5.4)	38 (17.1)	0.006
Pericardial effusion	20 (6.4)	5 (5.4)	15 (6.8)	0.662
Previous surgical therapy	68 (21.7)	37 (40.2)	31 (14.0)	<0.001

Data are shown as n (%) per each group, unless otherwise noted

### Prognostic factors predicting efficacy of pemetrexed

**Fig. 2:**
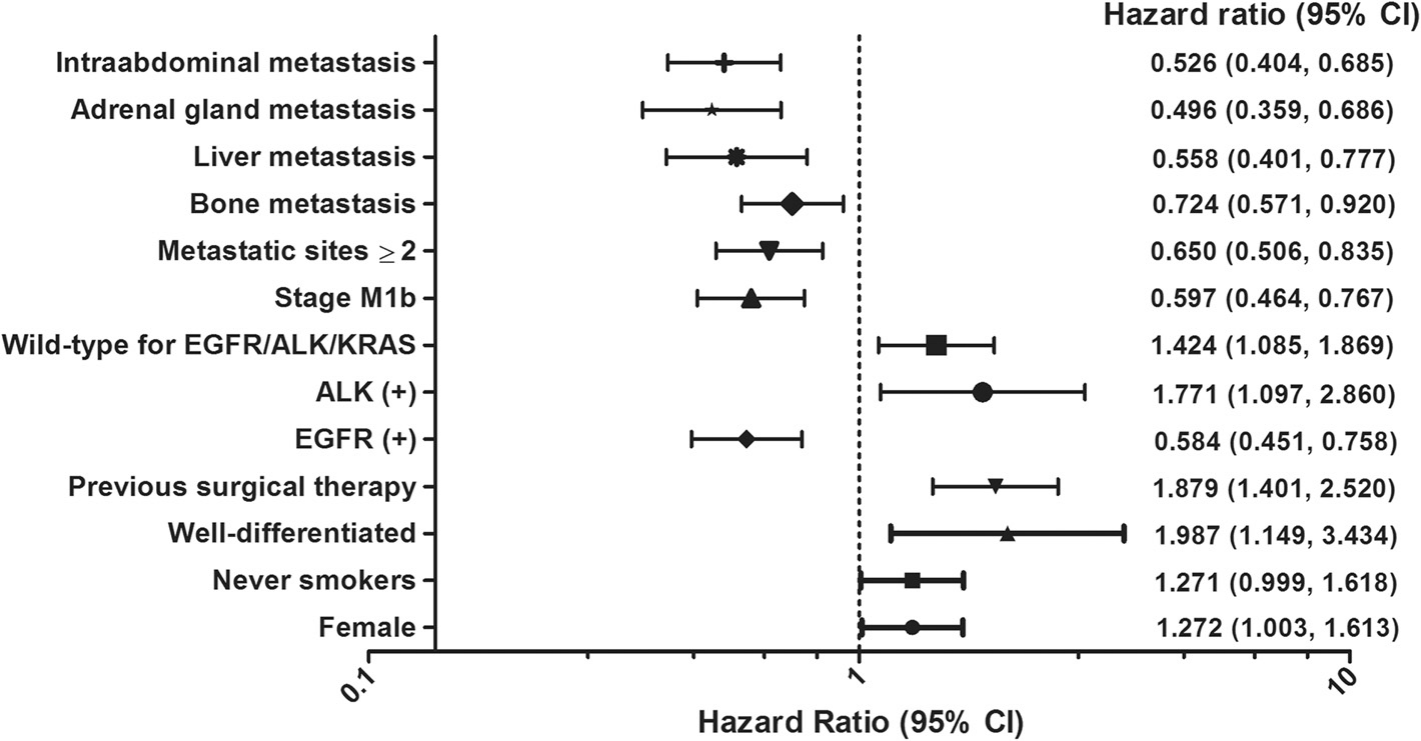
Prognostic factors by univariate analysis. The Forest plot of prognostic factors for non-small-cell lung cancer patients treated with pemetrexed-containing therapy. The Forest plot shows the hazard ratio and 95 % confidence interval of the prognostic factors by univariate analysis. *W/D* well-differentiated cancer; *EGFR* epidermal growth factor receptor; *ALK* anaplastic lymphoma kinase; *KRAS* Kirsten rat sarcoma viral oncogene homolog; *CI* confidence interval

When parameters were analyzed by univariate analysis (*P* <0.1), sex, smoking status, level of differentiation, history of previous surgical therapy, gene alteration, tumor burden and sites of metastasis were revealed as significant prognostic factor predicting efficacy of pemetrexed (Fig. 2). These parameters were put in multivariate analysis by Cox regression model. As a result, never smoking status (hazard ratio [HR], 1.997; 95 % confidence interval [CI], 1.442–2.766; *P* <0.001), *EGFR* mutation (HR, 0.520; 95 % CI, 0.377–0.718; *P* <0.001), presence of two or more metastatic sites (HR, 0.668; 95 % CI, 0.471–0.946; *P* = 0.023) and presence of intraabdominal metastatic lesion (HR, 0.590; 95 % CI, 0.410–0.847; *P* = 0.004) were independent prognostic factor predicting efficacy of pemetrexed containing regimen (Table 4).

**Table 4:** Prognostic factors predicting the efficacy of pemetrexed in patients with non-squamous non-small-cell lung cancer assessed using a multivariate Cox model

Characteristics	Hazard ratio (95 % CI)	*P*-value
Never-smoker	1.997 (1.442–2.766)	<0.001
Metastasis to intraabdomen	0.590 (0.410–0.847)	0.004
Number of metastatic lesion ≥2	0.668 (0.471–0.946)	0.023
Presence of *EGFR* mutation	0.520 (0.377–0.718)	<0.001

*CI* confidence interval; *EGFR* epidermal growth factor receptor

## Discussion

This is the first study to compare the characteristics of good responders and poor responders to pemetrexed therapy directly. Our current study findings demonstrated that smoking status, *EGFR* mutation, tumor burden and intra-abdominal metastasis are predictive factors for the response of these lung cancer patients to pemetrexed. In addition to *EGFR* mutation, molecular genetic factors, such as *ALK* translocation and wild-type for *EGFR/ALK/KRAS,* were more frequently seen in the good responder group.

An activating *EGFR* mutation has been reported in various studies not to confer a survival benefit for any chemotherapeutic regimen other than tyrosine kinase inhibitors, with a median PFS of 5.8 months as a first-line and of 4.1 months as second-line and beyond, including a small number of patients who had received pemetrexed [13]. In addition, a previous study which compared efficacy of pemetrexed in relation to gene mutation reported that *EGFR* mutation was not associated with survival gain [5]. Accordingly, our present study demonstrated that patients with *EGFR* mutations were unlikely to benefit from pemetrexed therapy. On the other hand, previous studies reported that treatment outcomes which included PFS and the response rate for pemetrexed in NSCLC cases with *ALK* translocation were better than the patients with a wild-type for *ALK* [5, 14, 15]. However, other previous studies did not report any benefit of pemetrexed in NSCLC patients with *ALK* translocation [7, 16]. Our current study findings suggest that *ALK* translocation may be an indicator for the response to pemetrexed therapy, although it was not demonstrated in multivariate analysis. Since only 3 patients in our current study had received crizotinib prior to pemetrexed therapy, we could not investigate whether crizotinib could affect on treatment outcome of pemetrexed. However, we could assume that pemetrexed could be considered as second-line or a good alternative to crizotinib in patients with *ALK* translocation.

The number of metastatic site was also prognostic factor predicting efficacy of pemetrexed. In our present study, a larger proportion of good responders experienced recurrence after surgical therapy compared with poor responders. Because the patients who had received surgical therapy underwent regular follow-up assessments using chest computer tomography, they tended to show a low tumor burden. Furthermore, the good responders had fewer distant metastatic lesions when the cancer recurred after surgical resection. Additionally, a prospective study by Sanchez et al. reported that the numbers of metastatic sites and lesions had prognostic relevance [17]. Tumor burden has exhibited prognostic relevance for survival irrespective of therapeutic agents, cytotoxic chemotherapy, and use of a tyrosine kinase inhibitor such as gefitinib [18–20]. Zhao et al., reported that cancer cells can tolerate chemotherapy and acquire more stemness under hypoxic conditions [21]. Additionally, cells expressing CD166, a specific membrane molecule expressed in cancer stem cells, exhibited a higher survival rate against pemetrexed under hypoxic conditions [21]. We assume that this may explain the poor outcomes in NSCLC patients with a higher tumor burden because disseminated tumors reflect both the rapid growth of cancer cells and greater number of cancer cells under relatively hypoxic conditions. Along with tumor burden, metastatic sites in our current patients were correlated with their prognoses. Similar to previous studies that demonstrated a correlation of metastases to the brain, bone and liver with a poor prognosis, our current data indicates that presence of intraabdominal metastasis significantly correlate with a poor response to pemetrexed [19, 22–24]. In addition, the metastatic lesion to liver, adrenal gland and bone also affected negatively on response to pemetrexed according to results by univariate analysis.

Lastly, never-smoking status was associated with good response to pemetrexed. This has been suggested in several previous studies, therefore, smoking status should be considered an important factor when choosing the treatment regimen [5, 7].

In the good responder group, 46.7 % of patients showed a partial response, while 53.3 % of patients exhibited stable disease. Achievement of a response is a robust marker of a biological therapeutic effect and correlates with both PFS and overall survival [25, 26]. Although the response rate was higher than the 9.1 % rate observed for overall pemetrexed monotherapy previously, it need to be noted that a large number of patients who have received pemetrexed for a long period of time without disease progression could not achieve a response [3].

The present study has several limitations. First, it had a retrospective design and patients did not receive treatment according to an established protocol which led to differences in the treatment regimens. Significantly more patients in the good responder group received pemetrexed in combination with other agent than the poor responder group (26.1 % versus 12.2 %, *P* = 0.002). However, the univariate analysis showed that the treatment option was not a prognostic factor (HR, 0.803; 95 % CI, 0.575–1.120; *P* = 0.196). In addition, Sun et al. reported that prior chemotherapeutic regiment and its response influence the efficacy of pemetrexed therapy [27]. As presented in Table 1, there was significant difference in response to gemcitabine and EGFR-tyrosine kinase inhibitor between two groups, while, the response of prior chemotherapeutic regimen did not affect on efficacy of pemetrexed according to univariate analysis. Second, we could not identify the exact performance status of each patient when they received pemetrexed. Many previous studies have reported that performance status represents an important prognostic factor, so it could also have affected the prognosis of our present cases [28–31]. Third, we compared two groups by extracting the top 5 % and the bottom 12 % of patients from the initial patient population. Because this was not an enumeration study, the study design itself could have introduced a bias. However, we selected the top 5 % since the section between approximately 5–6 % was sharply changing when patients arranged in order of duration of pemetrexed therapy (Fig. 1). To show a clear contrast between two groups, we selected two sections in the top and bottom 5 % although the proportion of poor responders increased to 12 % for their same amount of pemetrexed usage.

## Conclusions

In conclusion, factors such as smoking status, genetic alteration status, intra-abdominal metastasis, and tumor burden may be considered as predictive factors for the response to pemetrexed therapy in non-squamous NSCLC.

## Abbreviations

ALK, anaplastic lymphoma kinase; EGFR, epidermal growth factor receptor; HR, hazard ratio; KRAS, Kirsten rat sarcoma viral oncogene homolog; NSCLC, non-small-cell lung cancer; PFS, progression-free survival; SD, stable disease; TS, thymidylate synthase; TTF-1, thyroid transcription factor-1

## References

[1] Jung KW, Won YJ, Kong HJ, Oh CM, As S, Lee JS (2013). Survival of Korean Adult Cancer Patients by Stage at Diagnosis, 2006-2010: National Cancer Registry Study. Cancer Res Treat.

[2] Scagliotti GV, Parikh P, von Pawel J, Biesma B, Vansteenkiste J, Manegold C (2008). Phase III study comparing cisplatin plus gemcitabine with cisplatin plus pemetrexed in chemotherapy-naive patients with advanced-stage non-small-cell lung cancer. J Clin Oncol.

[3] Hanna N, Shepherd FA, Fossella FV, Pereira JR, De Marinis F, von Pawel J (2004). Randomized phase III trial of pemetrexed versus docetaxel in patients with non-small-cell lung cancer previously treated with chemotherapy. J Clin Oncol.

[4] Grønberg BH, Bremnes RM, Fløtten O, Amundsen T, Brunsvig PF, Hjelde HH (2009). Phase III study by the Norwegian lung cancer study group: pemetrexed plus carboplatin compared with gemcitabine plus carboplatin as first-line chemotherapy in advanced non-small-cell lung cancer. J Clin Oncol.

[5] Park S, Park TS, Choi CM, Lee DH, Kim SW, Lee JS (2015). Survival Benefit of Pemetrexed in Lung Adenocarcinoma Patients With Anaplastic Lymphoma Kinase Gene Rearrangements. Clin Lung Cancer.

[6] Solomon BJ, Mok T, Kim DW, Wu YL, Nakagawa K, Mekhail T (2014). First-line crizotinib versus chemotherapy in ALK-positive lung cancer. New Engl J Med.

[7] Shaw AT, Varghese AM, Solomon BJ, Costa DB, Novello S, Mino-Kenudson M (2013). Pemetrexed-based chemotherapy in patients with advanced, ALK-positive non-small cell lung cancer. Ann Oncol.

[8] Sun JM, Han J, Ahn JS, Park K, Ahn MJ (2011). Significance of thymidylate synthase and thyroid transcription factor 1 expression in patients with nonsquamous non-small cell lung cancer treated with pemetrexed-based chemotherapy. J Thorac Oncol.

[9] Grønberg BH, Lund-Iversen M, Strøm EH, Brustugun OT, Scott H (2013). Associations between TS, TTF-1, FR-α, FPGS, and overall survival in patients with advanced non–small-cell lung cancer receiving pemetrexed plus carboplatin or gemcitabine plus carboplatin as first-line chemotherapy. J Thorac Oncol.

[10] Mou W, Xue H, Tong H, Sun S, Zhang Z, Zhang C (2014). Prognostic value of serum leptin in advanced lung adenocarcinoma patients with cisplatin/pemetrexed chemotherapy. Oncol Lett.

[11] Zukin M, Barrios CH, Pereira JR, Ribeiro Rde A, Beato CA, Do Nascimento YN (2013). Randomized phase III trial of single-agent pemetrexed versus carboplatin and pemetrexed in patients with advanced non-small-cell lung cancer and Eastern Cooperative Oncology Group performance status of 2. J Clin Oncol.

[12] Eisenhauer EA, Therasse P, Bogaerts J, Schwartz LH, Sargent D, Ford R (2009). New response evaluation criteria in solid tumours: revised RECIST guideline (version 1.1). Eur J Cancer.

[13] Paz-Ares L, Soulieres D, Moecks J, Bara I, Mok T, Klughammer B (2014). Pooled analysis of clinical outcome for EGFR TKI-treated patients with EGFR mutation-positive NSCLC. J Cell Mol Med.

[14] Camidge DR, Kono SA, Lu X, Okuyama S, Baron AE, Oton AB (2011). Anaplastic lymphoma kinase gene rearrangements in non-small cell lung cancer are associated with prolonged progression-free survival on pemetrexed. J Thorac Oncol.

[15] Lee JO, Kim TM, Lee SH, Kim DW, Kim S, Jeon YK (2011). Anaplastic lymphoma kinase translocation: a predictive biomarker of pemetrexed in patients with non-small cell lung cancer. J Thorac Oncol.

[16] Shaw AT, Kim DW, Nakagawa K, Seto T, Crino L, Ahn MJ (2013). Crizotinib versus chemotherapy in advanced ALK-positive lung cancer. N Engl J Med.

[17] Sanchez De Cos Escuin J, Abal Arca J, Melchor Iniguez R, Miravel Sorribes L, Nunez Ares A, Hernandez Hemandez JR (2014). Tumor, node and metastasis classification of lung cancer--M1a versus M1b--analysis of M descriptors and other prognostic factors. Lung Cancer.

[18] Joss RA, Burki K, Dalquen P, Schatzmann E, Leyvraz S, Cavalli F (1990). Combination chemotherapy with mitomycin, vindesine, and cisplatin for non-small cell lung cancer. Association of antitumor activity with initial tumor burden and treatment center. Cancer.

[19] Oh Y, Taylor S, Bekele BN, Debnam JM, Allen PK, Suki D (2009). Number of metastatic sites is a strong predictor of survival in patients with nonsmall cell lung cancer with or without brain metastases. Cancer.

[20] Park JH, Kim TM, Keam B, Jeon YK, Lee SH, Kim DW (2013). Tumor burden is predictive of survival in patients with non-small-cell lung cancer and with activating epidermal growth factor receptor mutations who receive gefitinib. Clin Lung Cancer.

[21] Zhao M, Zhang Y, Zhang H, Wang S, Zhang M, Chen X (2015). Hypoxia-induced cell stemness leads to drug resistance and poor prognosis in lung adenocarcinoma. Lung Cancer.

[22] Fujimoto D, Ueda H, Shimizu R, Kato R, Otoshi T, Kawamura T (2014). Features and prognostic impact of distant metastasis in patients with stage IV lung adenocarcinoma harboring EGFR mutations: importance of bone metastasis. Clin Exp Metastasis.

[23] Riihimäki M, Hemminki A, Fallah M, Thomsen H, Sundquist K, Sundquist J (2014). Metastatic sites and survival in lung cancer. Lung Cancer.

[24] Wu KL, Tsai MJ, Yang CJ, Chang WA, Hung JY, Yen CJ (2015). Liver metastasis predicts poorer prognosis in stage IV lung adenocarcinoma patients receiving first-line gefitinib. Lung Cancer.

[25] Louvet C, de Gramont A, Tournigand C, Artru P, Maindrault‐Goebel F, Krulik M (2001). Correlation between progression free survival and response rate in patients with metastatic colorectal carcinoma. Cancer.

[26] Johnson KR, Ringland C, Stokes BJ, Anthony DM, Freemantle N, Irs A (2006). Response rate or time to progression as predictors of survival in trials of metastatic colorectal cancer or non-small-cell lung cancer: a meta-analysis. Lancet Oncol.

[27] Sun JM, Oh DY, Lee SH, Kim DW, Im SA, Kim TY (2010). The relationship between response to previous systemic treatment and the efficacy of subsequent pemetrexed therapy in advanced non-small cell lung cancer. Lung Cancer.

[28] Bittner N, Baliko Z, Sarosi V, Laszlo T, Toth E, Kasler M (2015). Bone Metastases and the EGFR and KRAS Mutation Status in Lung Adenocarcinoma - The Results of Three Year Retrospective Analysis. Pathol Oncol Res.

[29] Sculier JP, Chansky K, Crowley JJ, Van Meerbeeck J, Goldstraw P (2008). The impact of additional prognostic factors on survival and their relationship with the anatomical extent of disease expressed by the 6th Edition of the TNM Classification of Malignant Tumors and the proposals for the 7th Edition. J Thorac Oncol.

[30] Kawaguchi T, Takada M, Kubo A, Matsumura A, Fukai S, Tamura A (2010). Performance status and smoking status are independent favorable prognostic factors for survival in non-small cell lung cancer: a comprehensive analysis of 26,957 patients with NSCLC. J Thorac Oncol.

[31] Albain KS, Crowley JJ, LeBlanc M, Livingston RB (1991). Survival determinants in extensive-stage non-small-cell lung cancer: the Southwest Oncology Group experience. J Clin Oncol.

